# Thymoma‐Associated Systemic Lupus Erythematosus and Myasthenia Gravis

**DOI:** 10.1155/crrh/7670778

**Published:** 2026-04-16

**Authors:** Keysha González-Ramos, Ileana Rivera-Burgos, Carlos Cedeño, Jean Lafontaine, María J. Marcos-Martínez, Luis M. Vilá

**Affiliations:** ^1^ Division of Rheumatology, Department of Medicine, University of Puerto Rico School of Medicine, Medical Sciences Campus, San Juan, Puerto Rico, USA; ^2^ Division of Rheumatology, Department of Medicine, Hospital Damas, Ponce, Puerto Rico, USA; ^3^ Division of Hematology, Medical Oncology, Department of Medicine, University of Puerto Rico School of Medicine, Medical Sciences Campus, San Juan, Puerto Rico, USA; ^4^ Department of Pathology and Laboratory Medicine, University of Puerto Rico School of Medicine, Medical Sciences Campus, San Juan, Puerto Rico, USA, lhsc.on.ca

## Abstract

Thymomas, rare tumors originating from thymic epithelial cells, can disrupt immune tolerance and contribute to the development of autoimmune diseases. While thymomas are commonly associated with myasthenia gravis (MG), their link to autoimmune rheumatic diseases such as systemic lupus erythematosus (SLE) is less frequently observed. We present a case of a 32‐year‐old woman with SLE who was admitted with headache, blurred vision in her right eye with ptosis, generalized weakness, and dysphagia. She had been experiencing dysphagia and weakness for the past year, with symptoms intensifying in the three weeks leading up to admission. Her SLE had been diagnosed a year earlier, presenting with polyarthritis, positive antinuclear and anti‐dsDNA antibodies, and low levels of C3 and C4. Inpatient laboratories revealed positive anti‐acetylcholine receptor antibodies. A chest computed tomography (CT) scan revealed an anterior mediastinal mass invading the left pleural space as well as the left brachial and cephalic veins. A CT‐guided biopsy of the mass confirmed a thymoma consistent with World Health Organization type AB. She was diagnosed with MG and thymoma. MG was managed with prednisone, pyridostigmine, and intravenous immunoglobulin. She underwent four cycles of neoadjuvant chemotherapy for her thymoma, with two additional cycles, with a modified treatment regimen, administered to enhance tumor reduction prior to surgery. She underwent thymectomy, pericardiectomy, venorrhaphy, and pneumorrhaphy and was initiated on radiation therapy due to tumor invasion of adjacent structures. At 12‐month follow‐up, her MG symptoms had completely resolved. As for her SLE, arthralgias persisted in the absence of arthritis, and no additional lupus manifestations developed. This case illustrates the diagnostic challenges posed by overlapping autoimmune disorders and emphasizes the need for careful evaluation to identify underlying malignancies, like thymoma, in patients presenting with atypical or evolving autoimmune symptoms.

## 1. Introduction

Thymomas are uncommon tumors arising from thymic epithelial cells, affecting approximately 0.13–0.32 individuals per 100,000 each year [1]. They constitute the majority of anterior mediastinal masses encountered clinically, accounting for around 40% of mediastinal lesions [2]. These tumors are frequently linked to paraneoplastic autoimmune conditions, particularly myasthenia gravis (MG), which occurs in 30%–50% of thymoma patients, while 10%–15% of individuals with MG harbor thymomas [2]. The association with SLE is much less common, occurring in only about 1.5% of cases [3]. A 15‐year retrospective multicenter study of patients supports the coexistence of SLE and thymic epithelial tumors (TET) [4]. Furthermore, a recent Mendelian randomization study suggests a two‐way causal relationship between MG and SLE, underscoring the clinical importance of their co‐occurrence [5]. Although such associations are rare, they may remain undetected due to the slow‐growing and often silent nature of thymomas. Herein, we present the case of a young woman initially diagnosed with SLE, who later developed MG, leading to further evaluation that uncovered an underlying thymoma.

## 2. Case Presentation

A 32‐year‐old woman with SLE was hospitalized, presenting with a headache, blurred vision in her right eye with ptosis, generalized weakness, and dysphagia. The headache began three days prior to admission, localized to the right retro‐orbital area and accompanied by blurred vision. She had been experiencing dysphagia and weakness for the past year, with symptoms intensifying in the three weeks leading up to admission.

SLE had been diagnosed a year earlier, initially presenting with polyarthritis, positive antinuclear (1:640, homogenous pattern), anti‐dsDNA and anti‐Ro antibodies, and C3 and C4 hypocomplementemia. There was no renal, pulmonary, or central nervous system involvement. Treatment included prednisone 10 mg daily, hydroxychloroquine 200 mg daily, and mycophenolate mofetil 500 mg twice daily.

On admission, her temperature was 36.2°C, heart rate was 88 beats per minute, blood pressure was 111/69 mm of mercury, respiratory rate was 16 breaths per minute, and oxygen saturation was 99% on room air. Right palpebral ptosis and proximal muscle weakness were noted in the upper and lower extremities during examination. No rash, alopecia, mucosal ulcers, Raynaud’s phenomenon, joint tenderness, or swelling was observed. The remainder of the physical examination was unremarkable.

### 2.1. Investigations

As shown in Table 1, the complete blood count, comprehensive metabolic panel, urinalysis, creatine phosphokinase, aldolase, erythrocyte sedimentation rate, and C‐reactive protein were all within normal limits. Immunologic testing results are detailed in Table 2, revealing positive antinuclear, anti‐dsDNA, anti‐Ro, anti‐Mi‐2, and antiacetylcholine receptor antibodies, along with low complement levels (C3 and C4 hypocomplementemia).

**TABLE 1:** General laboratory investigations during hospitalization.

Test	Result	Reference
White blood cell count	10,330/μL	4500–11,000/μL
Hemoglobin	15.0 g/dL	12–15 g/dL
Platelet count	310,000/μL	150,000–450,000/μL
Lymphocyte count	2640/μL	1500–4800/μL
Serum creatinine	0.5 mg/dL	0.6–1.2 mg/dL
Aspartate aminotransferase	22 IU/L	0–40 IU/L
Alanine aminotransferase	19 IU/L	0–40 IU/L
Creatine phosphokinase	65 U/L	30–145 U/L
Aldolase	8.0 U/L	1–7.5 U/L
Urinalysis		
Protein	30 mg/dL	Negative
White blood cells	16–25/Hpf	0–4/Hpf
Red blood cells	4–10/Hpf	0–3/Hpf
Casts	None	None
Erythrocyte sedimentation rate	25 mm/hr	0–20 mm/hr
C‐reactive protein	2.3 mg/L	0.3–5.0 mg/L

Abbreviation: Hpf, high power field.

**TABLE 2:** Immunologic laboratory work‐up conducted during hospitalization.

Test	Result	Reference
Lupus panel		
ANA	1:160 (homogenous)	Negative dilution
Anti‐dsDNA Ab	1: 160	Negative dilution
Anti‐Smith/ribonucleoprotein Ab	< 0.2 AI	< 1.0 AI
C3	71 mg/dL	90–180 mg/dL
C4	6.6 mg/dL	15–57 mg/dL
Anti‐Ro Ab	3.1 U/mL	< 1.0 U/mL
Anti‐La Ab	< 0.20 U/mL	< 1.0 U/mL
Antiphospholipid syndrome panel		
Lupus anticoagulant	Negative	Negative
Anti‐cardiolipin (IgA, IgG, IgM)	Negative	Negative
Anti‐β2‐glycoprotein I (IgA, IgG, IgM)	Negative	Negative
Myositis panel		
Anti‐Mi‐2 Ab	11 SI	< 11 SI
Anti‐synthetase Ab (Jo‐1, Ej, OJ, PL‐7, PL‐12)	< 11 SI	< 11 SI
Anti‐SRP Ab	< 11 SI	< 11 SI
Anti‐MDA5 Ab	< 11 SI	< 11 SI
Anti‐TIF‐1*γ* Ab	< 11 SI	< 11 SI
Anti‐NXP‐2 Ab	< 11 SI	< 11 SI
Myasthenia gravis tests		
Anti‐acetylcholine receptor Ab		
Receptor binding Ab	122.8	< 0.3 nmol/L
Receptor modulating Ab	95%	< 32% inhibition
Receptor blocking Ab	39%	< 15% inhibition

*Note:* ANA: antinuclear antibodies; Ab: antibodies; NXP‐2: nuclear matrix protein 2.

Abbreviations: MDA‐5, melanoma differentiation‐associated gene 5; SRP, signal recognition particle; TIF‐1*γ*, transcriptional intermediary factor 1‐gamma.

**FIGURE 1:**
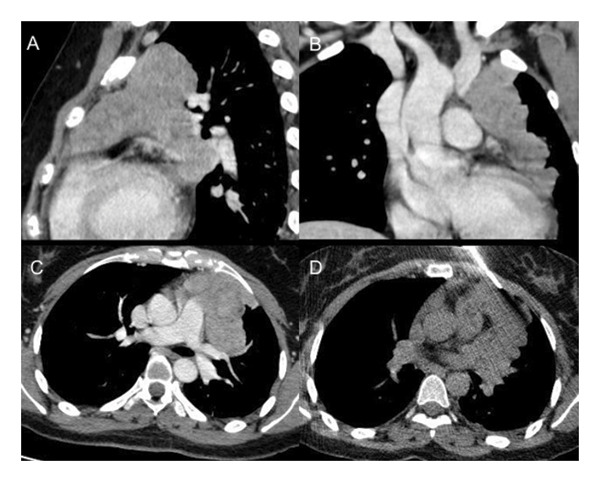
Chest computed tomography with and without contrast. (A) Sagittal, (B) coronal, and (C) axial views demonstrate a lobulated, solid, enhancing mass in the anterior mediastinum measuring 8.7 × 6.0 × 8.8 cm. Anteriorly, it is in contact with the chest wall. Posteriorly, the mass extends into the posterior mediastinum and abuts the left superior pulmonary vein, main pulmonary artery, and left atrial appendage. Superiorly, there is mass effect and evidence of invasion into the left brachiocephalic vein. A left pleural drop metastasis is also noted. (D) Axial view from a noncontrast CT performed during core needle biopsy.

**FIGURE 2:**
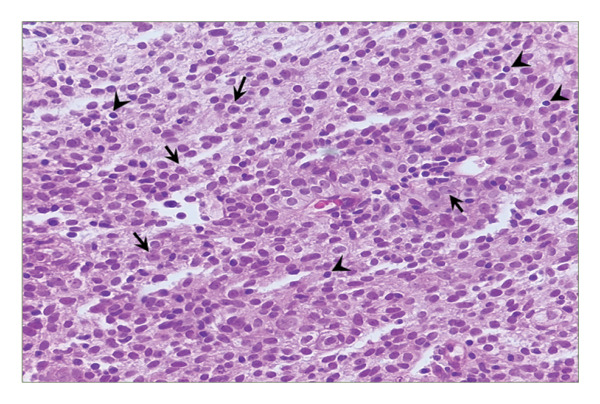
Thymic mass computed tomography‐guided core biopsy. High‐power magnification (400x) of a hematoxylin and eosin‐stained section shows a predominant population of bland, oval to spindled thymic epithelial cells (black arrows) with small to inconspicuous nucleoli and open nuclear chromatin, intermixed with a dense infiltrate of small lymphocytes (black arrowheads).

**FIGURE 3:**
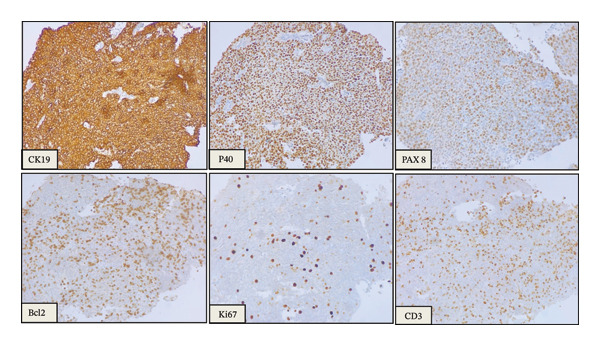
Immunohistochemical profile (200X). CK19 (membranous), p40 (nuclear), PAX8 (nuclear), and Bcl‐2 are positive in ovoid to spindle epithelial cells. There is a low proliferation index, as indicated by the Ki‐67 (MIB). Note the dense intermixed infiltration of CD3‐positive small, mature T‐lymphocytes.

Computed tomography (CT) imaging revealed an anterior mediastinal mass with invasion of the left pleural space and the left brachiocephalic vein (Figure 1). These findings raised concern for an underlying neoplastic process, prompting a CT‐guided core needle biopsy. Histopathologic evaluation of the biopsy specimen (Figure 2) and immunohistochemical staining (Figure 3), confirmed the diagnosis of a type AB thymoma, according to the World Health Organization (WHO) classification.

### 2.2. Treatment and Outcomes

Regarding her SLE, hydroxychloroquine was discontinued due to its potential to exacerbate MG symptoms. For her MG, she received high‐dose prednisone at 40 mg daily, and the mycophenolate mofetil dose was increased to 1000 mg twice daily. In addition, during the hospitalization, she underwent a five‐day course of intravenous immunoglobulin therapy and was started on pyridostigmine, titrated to 60 mg four times daily to optimize symptom control. Following clinical stabilization of her MG, she was discharged with a treatment plan in place for outpatient management of her thymoma.

Two months later, she was started on cyclophosphamide 500 mg/m^2^, doxorubicin 50 mg/m^2^, and cisplatin 50 mg/m^2^ administered intravenously every three weeks. Mycophenolate mofetil was reduced to 500 mg daily to avoid myelosuppression. After the first cycle, there was a complete resolution of dysphagia and extremity weakness. She completed a total of four cycles. Follow‐up chest imaging, four months later, showed the mass continued to abut the distal right ventricular outflow tract. As a result, the treatment regimen was modified to carboplatin and paclitaxel for two cycles, resulting in shrinkage of the mass better for resection. Four months later she underwent thymectomy, pericardiectomy, venorrhaphy, and pneumorrhaphy, and was initiated on radiation therapy.

At the twelve‐month follow‐up, when evaluating her SLE, arthralgias have persisted without arthritis, but no additional clinical manifestations of SLE had developed. Repeated serologies revealed a normal C3 level with a low C4 level and persistently elevated anti‐dsDNA antibodies. Oral prednisone had been gradually tapered to 7.5 mg daily. Mycophenolate mofetil continued at 500 mg daily. In terms of her MG, there was resolution of headaches, blurry vision, palpebral ptosis, generalized weakness, and dysphagia. She continued on pyridostigmine 60 mg four times daily.

## 3. Discussion

We report a case of thymoma‐associated paraneoplastic syndrome manifesting as both SLE and MG. Although SLE was diagnosed one year prior to the discovery of the thymoma, this temporal relationship does not necessarily establish causality. It remains possible that a preclinical or evolving systemic autoimmune process was already underway or that the thymoma was present but undetected at the time of lupus onset. Thymomas often exhibit indolent clinical behavior, remaining asymptomatic for years and frequently discovered incidentally on imaging [6], yet they may manifest through paraneoplastic syndromes that serve as early indicators of malignancy. In a study of 31 patients with available prior imaging, 77% had a thymoma present 1 to 26 years earlier, with an average of 6 years [7]. One case documented the slow growth of thymoma over 21 years. This indolent nature is further supported by the long‐time intervals between treatment and recurrence, which ranged from 1 to 32 years, with an average of 5 years. Despite their generally slow progression, thymomas can metastasize to the pleura, pericardium, and other distant sites [7]. Although often indolent, the potential for local invasion or distant spread highlights the importance of early recognition and thorough evaluation in patients with suggestive or evolving autoimmune symptoms.

The thymus plays a crucial role in nurturing cell‐mediated immunity and ensuring self‐tolerance. Unlike thymic follicular hyperplasia, thymomas are characterized by aberrant T‐cell maturation and lack intact autoantigen presentation. Recent studies suggest that these tumors may export improperly selected, autoantigen‐specific T cells into the peripheral circulation, thereby contributing to systemic autoimmune phenomena [8]. MG‐associated thymomas, in particular, demonstrate disordered intratumorous T‐cell development, leading to the generation of autoantigen‐reactive T cells—predominantly of the CD8+ subset—which are subsequently released into the periphery and implicated in autoimmune pathogenesis. Flow cytometry analyses have revealed a significant increase in circulating naïve CD45RA + CD8+ T cells in patients with thymoma, a finding that diminishes following thymectomy, further supporting the hypothesis that thymomas alter the peripheral T‐cell repertoire. The interplay between peripherally exported autoantigen‐specific CD4+ and CD8+ T cells and B cells appears to initiate and perpetuate MG, particularly in patients exhibiting aberrant CD4+ T‐cell responses, consistent with a two‐step model of thymoma‐induced autoimmunity. These findings underscore the central role of thymoma‐driven T‐cell dysregulation in the development of paraneoplastic autoimmunity, offering insight into the underlying mechanisms linking TET with systemic autoimmune diseases such as MG and, more rarely, SLE.

The relationship between SLE and TET has been the subject of growing interest due to the potential for paraneoplastic syndromes in these patients. A French observational study by Noel et al. analyzed the clinical, biological, and pathological characteristics of patients with both SLE and TET in a retrospective multicenter series [4]. Patients with this association commonly exhibited SLE symptoms such as polyarthralgia, pericarditis, myositis, photosensitivity, glomerulonephritis, and autoimmune hemolytic anemia, which appeared before or after the TET diagnosis. Another study by Bernard et al. presented the characteristics of 37 thymoma‐associated SLE patients, including two of their own cases and 35 additional cases from the literature [9]. Among these patients, 78% were female, the median age at tumor diagnosis was 55 years, and the median age at SLE diagnosis was 53 years. A third of SLE patients (32%) were diagnosed before the thymoma (average interval of 2 years), while 35% had both conditions diagnosed simultaneously, and 32% developed SLE after thymoma diagnosis (average interval of 4 years). The clinical features of SLE were typical, with joint involvement in 59%, skin involvement in 35%, and serositis in 38%. Our patient was female and presented with polyarthritis one year prior to her diagnosis of thymoma, consistent with the symptoms observed in the aforementioned studies.

Although our patient did not present with clinical symptoms consistent with dermatomyositis, anti‐Mi2 antibodies were elevated. Detection of myositis‐specific antibodies in patients with coexisting inflammatory myositis and MG is inconsistent; in one series, such antibodies were often absent, with only one of four patients showing a weakly positive PL‐7 antibody [10]. Other reports found anti‐Jo‐1 and Ro‐52 antibodies in patients with MG and inflammatory myopathy, while some cases lacked myositis‐specific antibodies entirely [11]. To our knowledge, anti‐Mi2 antibodies have not previously been reported in association with MG or thymoma.

Treating patients with both SLE and MG is challenging due to concerns over antimalarial medications. Chloroquine, for example, has been associated with reversible myasthenia‐like symptoms. A retrospective study by Jallouli et al. focused on hydroxychloroquine use and reviewed existing literature. Of the eight patients who developed MG after starting hydroxychloroquine (median time of 44 months), four had the drug discontinued, but three did not see improvement in their myasthenic symptoms [12]. It remains unclear whether treatment for one condition may mask flare‐ups of the other or if hydroxychloroquine poses a risk in these patients. In our case, following a multidisciplinary discussion, the decision was made to discontinue the medication.

## 4. Conclusion

This case highlights the rare coexistence of thymoma, SLE, and MG, emphasizing the need for clinicians to consider overlapping autoimmune conditions. It also suggests that the thymoma could have been present from the beginning but was previously missed, underscoring the importance of recognizing unusual symptoms that may indicate an underlying thymoma. Early identification through imaging and biopsy can significantly improve diagnosis, management, and prognosis. Treatment requires a multidisciplinary approach that balances immunosuppression with the management of associated complications.

## Funding

This research did not receive any specific grant from funding agencies in the public, commercial, or not‐for‐profit sectors.

## Consent

Written informed consent for publication was obtained from the patient.

## Conflicts of Interest

The authors declare no conflicts of interest.

## Data Availability

Data can be obtained from the corresponding author upon request.
